# Novel algorithms for LDD motif search

**DOI:** 10.1186/s12864-019-5701-6

**Published:** 2019-06-06

**Authors:** Peng Xiao, Martin Schiller, Sanguthevar Rajasekaran

**Affiliations:** 1 Department of Computer Science and Engineering, University of Connecticut, 371 Fairfield Road, Storrs, 06269 CT USA; 2 School of Life Sciences, University of Nevada, Las Vegas, NV USA

**Keywords:** Motif search, Radix sort, Neighborhood tree

## Abstract

**Background:**

Motifs are crucial patterns that have numerous applications including the identification of transcription factors and their binding sites, composite regulatory patterns, similarity between families of proteins, etc. Several motif models have been proposed in the literature. The (*l,d*)-motif model is one of these that has been studied widely. However, this model will sometimes report too many spurious motifs than expected. We interpret a motif as a biologically significant entity that is evolutionarily preserved within some distance. It may be highly improbable that the motif undergoes the same number of changes in each of the species. To address this issue, in this paper, we introduce a new model which is more general than (*l,d*)-motif model. This model is called (*l,d*_1_,*d*_2_)-motif model (LDDMS) and is NP-hard as well. We present three elegant as well as efficient algorithms to solve the LDDMS problem, i.e., LDDMS1, LDDMS2 and LDDMS3. They are all exact algorithms.

**Results:**

We did both theoretical analyses and empirical tests on these algorithms. Theoretical analyses demonstrate that our algorithms have less computational cost than the pattern driven approach. Empirical results on both simulated datasets and real datasets show that each of the three algorithms has some advantages on some (*l,d*_1_,*d*_2_) instances.

**Conclusions:**

We proposed LDDMS model which is more practically relevant. We also proposed three exact efficient algorithms to solve the problem. Besides, our algorithms can be nicely parallelized. We believe that the idea in this new model can also be extended to other motif search problems such as Edit-distance-based Motif Search (EMS) and Simple Motif Search (SMS).

## Background

Motif search has many applications in solving some crucial biological problems. For example, finding DNA motifs is very important for the determination of open reading frames, identification of gene promoter elements, location of RNA degradation signals, and the identification of alternative splicing sites [1, 2]. For more than 15 years, motif search has stimulated a lot of interest from researchers in different areas.

There are many models of motif search. One popular model that has been studied extensively is the (*l,d*)-motif model. The corresponding motif search problem is called LDMS. The input for the LDMS problem consists of *n* input sequences each of length *m*, and two integers *l* and *d*. The task is to find all the strings (also called (*l,d*)-motifs) of length *l* each that occur in each of the input sequences within a hamming distance of *d*. The LDMS problem is known to be NP-hard [3, 4].

Motifs can be thought of as evolutionarily preserved biological information. This information might have undergone different changes in different species. The (*l,d*)-motif model captures this possibility by requiring that the motif occur within a hamming distance of *d* in **each** sequence. However, this requirement may be more stringent than needed. When some biological information undergoes changes (e.g., mutations) in various species, the amount of change may not be the same across all the species. Some might have undergone more changes than the others. If we think of *d* as an upper bound on the amount of change, then it is conceivable (and very likely) that some of the species have undergone less changes. As a result, the (*l,d*)-motif model is likely to admit many spurious strings as motifs. These strings might occur by random chance and get qualified as motifs. Because of this, the LDMS algorithms might take longer time than actually needed. To rectify these shortcomings, in this paper we propose a new model of motifs. This model is called (*l,d*_1_,*d*_2_)-model. The corresponding motif search problem is called the LDDMS problem and defined next.

### **Definition 1**

The input for the LDDMS problem has *n* biological sequences each of length *m* and three integers *l,d*_1_, and *d*_2_. The problem is to find all the strings *M* of length *l* that have the following two properties: 1) *M* should occur in each of the *n* input strings within a hamming distance of *d*_1_. This requirement is referred to as the (*l,d*_1_)-condition; and 2) *M* should occur in at least one of the *n* input strings within a hamming distance of *d*_2_. This requirement is referred to as the (*l,d*_2_)-condition.

### Validity of the (*l,d*_1_,*d*_2_)-motif model

In this section we demonstrate the validity of the (*l,d*_1_,*d*_2_)-motif model with a simple random model for mutations. Assume that the species under consideration have the same origin. Let *M* be an original motif of length *l*. Consider a random model where the number of mutations occurring in the species is uniformly distributed in the range $\left [ 0,\frac {l}{2}\right ]$. Let *n* be the number of species and let the number of mutations that have occurred in these species be *X*_1_,*X*_2_,…,*X*_*n*_, respectively and let *Y*= min{*X*_1_,*X*_2_,…,*X*_*n*_} and *Z*= max{*X*_1_,*X*_2_,…,*X*_*n*_}. It is easy to show that:

$$\begin{array}{*{20}l} E[Y] = \sum\limits_{k=1}^{l/2} k \frac{(l/2-k+1)^{n} - (l/2-k)^{n}}{(l/2+1)^{n}} \end{array} $$

$$\begin{array}{*{20}l} E[Z] = \frac{1}{(l/2+1)^{n}} \sum\limits_{k=1}^{l/2}[(k+1)^{n}-k^{n}] \end{array} $$

Thus the difference between *Y* and *Z* could be quite large! As an example consider an input of 20 sequences, each of length 600 and let *l*=10. Assume that the number of mutations *d* is uniformly random in the range [0,5]. If we set *d*_2_=1, the probability that there exists at least one DNA sequence such that the motif occurs with a hamming distance of at most *d*_2_ is:

$$\begin{array}{*{20}l} p = 1 - {\left(\frac{4}{6}\right)}^{20} \approx 0.9997 \end{array} $$

When *n* is larger than 20, this probability will become even higher. Therefore, it is quite reasonable to add the (*l,d*_2_)-condition into the LDMS model.

It is easy to see that if $d_{2} \geqslant d_{1}$, then the (*l,d*_2_)-condition becomes trivial and the LDDMS problem will become the standard LDMS problem. Thus, the LDMS problem is a special case of the LDDMS problem. If *d*_2_=0, it means that we want to look for a motif that appears exactly in at least one of the input sequences. In the rest of this paper we assume that *d*_2_<*d*_1_.

### Related work

(*l,d*) motif search is also referred to as Planted Motif Search (PMS) problem in some literature. Since (*l,d*_1_,*d*_2_) motif search is closely related to PMS and we will use a PMS solver in one of the LDDMS algorithms, it is necessary to discuss some of the latest PMS algorithms.

In 2012, Yu, et al., proposed PairMotif to solve PMS problems [5]. They reduced the size of candidate motifs and scanned *l*-mers by selecting pairs of *l*-mers from different input sequences and then generate the common neighbors. The authors tested PairMotif algorithm on simulated data as well as on five real data sets from [6], which are preproinsulin, DHFR, c-fos, metallothionein and Yeast ECB. It can solve the weak instance (27, 9) within 10 hours. They also showed that PairMotif is more stable in solving PMS problem in longer input sequences [5].

Sometimes, biologists may also be interested in motifs that occur in a fraction of the input strings. The problem of identifying such motifs is known as quorum Planted Motif Search (qPMS). In this case, in addition to *l* and *d* and *n* strings there is an extra input parameter *q*. The problem is to identify all the (*l,d*,*q*)-motifs, that is, all the (*l,d*)-motifs that occur in at least *q*% of the input strings. In 2014, Tanaka proposed TraverStringRef in [7]. This algorithm is based on the PMS8 algorithm of Nicolae and Rajasekaran [8]. This is the first algorithm that solved the challenging DNA instance with (*l,d*,*q*)=(25,10,20) in a reasonable amount of time.

In 2015, Nicolae and Rajasekaran proposed qPMS9 [9]. It can solve challenging instances up to (25,10) using a single core machine and up to (30,13) using a 48-core machine. The algorithm is based on PMS8 proposed by the same authors [8], but it added quorum support and also included better pruning techniques to significantly reduce the size of the search space.

In 2016, Xiao, Pal and Rajasekaran proposed qPMS10 [3, 4]. qPMS10 is a randomized algorithm based on the idea of random sampling. It will first utilize any existing PMS solver on a subset of the input. Then the candidate motifs are filtered to get the correct motifs for the original problem. Probability analysis shows that with high probability, the result is correct. Experimental result shows that this algorithm is competitive especially when the dataset is large.

Not only mutations, but also insertions and deletions are important as they may also play critical roles in divergence of biological sequences [10, 11]. In this case, edit distance instead of hamming distance should be considered [12, 13]. This corresponding problem is modeled as *Edit-distance-based Motif Search (EMS)* problem. There are also some works in the literature on EMS (see e.g., [1, 12–15], and so on).

However, as far as the authors know, no such generalizations of PMS model exist in the published literature. Therefore, we propose LDDMS model and the corresponding algorithms.

## Methods

Since the LDMS problem is NP-hard, the LDDMS problem is also NP-hard. All the known exact algorithms for solving the LDMS problem take time that is exponential in some of the underlying parameters. In this paper, we present three efficient algorithms for solving the LDDMS problem. These algorithms are referred to as LDDMS1, LDDMS2 and LDDMS3. Time complexities of these three algorithms are analysed. Experimental results on simulated dataset and real datasets both demonstrate that our algorithms are efficient.

### Description of LDDMS algorithms

For any *l*-mer *u* we define its *d*-friendhood as the set of *l*-mers *v* whose hamming distance is exactly *d* from *u*; define its *d*-neighborhood as the set of *l*-mers *v* whose hamming distance is at most *d* from *u*.

For all the LDDMS algorithms, the input is a database *S* containing *n* sequences, each of length *m*, and integers *l*, *d*_1_ and *d*_2_; the output is all the strings of length *l* that meet both (*l,d*_1_)-condition and (*l,d*_2_)-condition.

A straight-forward solution is the pattern driven approach. If *Σ* is the alphabet under concern, there are |*Σ*|^*l*^ possible *l*-mers. For every such *l*-mer, check if it meets both the (*l,d*_1_)-condition and the (*l,d*_2_)-condition. If so, output this *l*-mer. Obviously, this algorithm takes too much time.

In addition to pattern driven approaches, we also have sample driven approaches. We could employ the following two step algorithm: 1) First find all the motifs that satisfy the (*l,d*_1_)-condition. This can be done using any of the LDMS algorithms. Let *C*_1_ be the set of these motifs; and 2) For every motif *x*∈*C*_1_, check if *x* satisfies the (*l,d*_2_)-condition and if so output *x*. We call this algorithm LDDMS1. Since qPMS9 is currently the most efficient LDMS algorithm [9], we will take advantage of it in LDDMS1 (See Algorithm 1).

Equivalently, we can also find (*l,d*_2_)-motifs in the first step, and then for every such motif check if it satisfies the (*l,d*_1_)-condition. We refer to this algorithm as LDDMS2 (See Algorithm 2).

Note that each valid motif has at least one *d*_2_-neighbor in at least one of the input sequences. We generate *n*(*m*−*l*+1)*l*-mers from each of the input sequences. *d*_2_-neighborhood of an *l*-mer *u* can be found by constructing the neighborhood tree. With *u* being the root and the height of the tree being *d*_2_, the level of a node is the hamming distance between *u* and this node. All the nodes of this tree, including the root and the leaves, will constitute the *d*_2_-neighborhood of *u*. In Step 3 of LDDMS2, we can employ radix sort and eliminate duplicates. In Step 4 the output *O*_2_ of valid motifs found will be in sorted order.


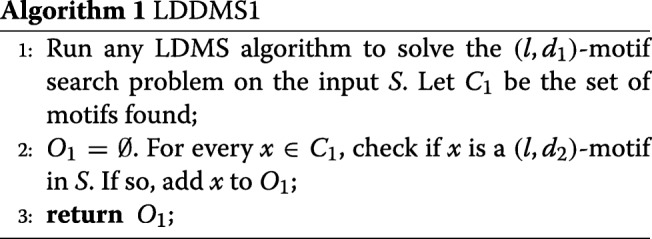



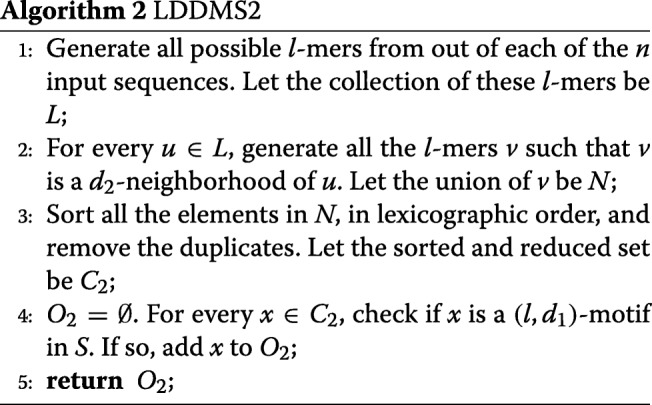


If *d*_2_ is very small (for example, *d*_2_ = 0 or 1), we can expect LDDMS2 to run faster than LDDMS1. This is because the *d*_2_-neighborhhod for any *l*-mer will be small. However, when *d*_2_ is large, the neighborhood tree will be large and so will be the number of candidate motifs. Therefore, LDDMS2 takes much more time and memory when *d*_2_ is large. To save time, one idea is to check the candidate motifs concurrently while constructing the neighborhood tree. During the checking process, some pruning conditions can be developed such that once certain conditions hold, a node is not explored deeper. The stronger the pruning condition is, the faster the algorithm will be. Inspired by similar pruning ideas proposed for the LDMS model [16], we develop LDDMS3 (See Algorithm 3).

#### **Definition 2**

Given an *l*-mer *u* from Sequence *i* (*i*∈ [1,*n*]), construct its *d*_2_-neighborhood tree. Let *x* be any node in this tree, denote *δ*(*x,i*,*q*) as the smallest hamming distance between *x* and any *l*-mer out of Sequence *q*. Denote *δ*(*x,i*,*I*) to be the maximum of *δ*(*x,i*,*q*) where *q*=1,2,...,*n* and *q*≠*i*.

$$\delta(x, i, I) = \max\limits_{q = 1, q \neq i}^{n} \delta(x, i, q) = \max \limits_{q = 1, q \neq i}^{n} \min \limits_{v \triangleleft_{l} s_{q}} Hd(v, x) $$

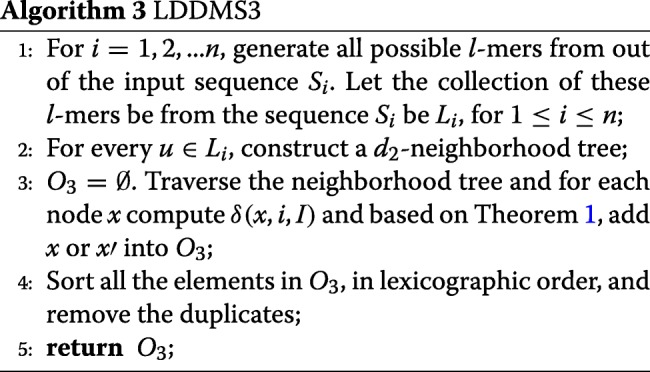


If *v* is an *l*-mer in the sequence *S*_*q*_, we denote it as: *v⊲*_*l*_*s*_*q*_. Also, *Hd*(*v,x*) is the hamming distance between *v* and *x*. By computing *δ*(*x,i*,*I*), we have the following pruning conditions [16].

#### **Theorem 1**

Traverse the *d*_2_-neighborhood tree of *u* in a depth-first manner and compute *δ*(*x,i*,*I*) where *x* is a node in the tree, *h* is the level of *x* (root is at level 0);

1. If *δ*(*x,i*,*I*)≤*d*_1_, output *x*;
2. If *δ*(*x,i*,*I*)−*d*_1_>*d*_2_−*h*, prune all the descendants from *x*;
3. If *δ*(*x,i*,*I*)−*d*_1_=*d*_2_−*h*, consider only *x*′ such that *x*′ is a child of *x* and *δ*(*x*′,*i,I*)=*δ*(*x,i*,*I*)−1;
4. If *δ*(*x,i*,*I*)−*d*_1_=*d*_2_−*h*−1, consider only *x*′ such that *x*′ is a child of *x* and *δ*(*x*′,*i,I*)≤*δ*(*x,i*,*I*).

### Analysis of LDDMS algorithms

#### Candidate size and expected number of motifs

In this section, we estimate candidate sizes of LDDMS1 and LDDMS2, i.e., |*C*_1_| and |*C*_2_|, and also the expected number of motifs that would be found. Such estimation is useful in computing the time complexities of these two algorithms.

Recall that in the benchmark dataset all the characters are generated from i.i.d. and there are *n* sequences with length *m* each. Given an *l*-mer *M*, the number of *l*-mers that have a hamming distance of ≤*d*_1_ from *M* is:

1 $$ N(\Sigma, l, d_{1}) = \sum\limits_{i=0}^{d_{1}} {{l}\choose{i}} (|\Sigma|-1)^{i}  $$

where *Σ* is the alphabet under concern.

The probability that a randomly chosen *l*-mer has a hamming distance of at most *d*_1_ from *M* is:

2 $$ p_{1}=\frac{N(\Sigma, l, d_{1})}{|\Sigma|^{l}}  $$

The probability that in a sequence of length *m*, there is at least one string *u* such that *u* and *M* are within a hamming distance of *d*_1_ is:

3 $$ p_{2}= 1-(1-p_{1})^{m-l+1}  $$

The probability that a randomly chosen *l*-mer occurs within a hamming distance of *d*_1_ in each of the *n* input sequences, each of length *m* is:

4 $$ p_{3}= p_{2}^{n}\\  $$

Therefore, the expected number of (*l,d*_1_)-motifs is:

5 $$ |C_{1}|= | \Sigma |^{l} p_{3}  $$

Similarly, the probability that a randomly chosen *l*-mer has a hamming distance of at most *d*_2_ from *M* is:

6 $$ p_{4}= \frac{ {\sum\nolimits}_{i=0}^{d_{2}} {{l}\choose{i}} (|\Sigma|-1)^{i}}{| \Sigma |^{l}}  $$

The probability that in a sequence of length *m*, there is at least one string *u* that has a hamming distance of at most *d*_2_ from *M* is:

7 $$ p_{5}= 1 - \left(1 - p_{4} \right)^{m - l + 1}  $$

Therefore, the expected number of (*l,d*_2_)-motifs is:

8 $$ |C_{2}|= | \Sigma |^{l} \left(1 - (1- p_{5})^{n}\right)  $$

In all of the above assertions we have assumed that the *l*-mers of a sequence are independent. Clearly, this is incorrect. However, such analyses have proven useful in estimating the number of motifs in practice (see e.g., [17]). Along these lines, let us look at the expected number of motifs that will be found, i.e., |*O*_1_| or |*O*_2_|. Let *M* be a random *l*-mer, *A*_*i*_ be the event that *M* has a neighbor that is within a hamming distance of *d*_2_ in exactly *i* of the input sequences. Similarly, let *B*_*j*_ be the event that *M* has a neighbor that is within a hamming distance of (*d*_2_,*d*_1_] in exactly *j* of the input sequences. It should be noted here that if *M* has a neighbor whose hamming distance is at most *d*_2_ in an input sequence, then it automatically will also have a neighbor that is within a hamming distance of *d*_1_ in such sequence since we assume *d*_2_<*d*_1_.

We want to know the probability that events *A*_*i*_ and *B*_*n*−*i*_ both happen, which means in each of the *n* input sequences, there is an *l*-mer that is within a hamming distance of *d*_2_ from *M* and also, in each of the remaining *n*−*i* input sequences, there will be an *l*-mer that is within a hamming distance of (*d*_2_,*d*_1_] from *M*.

Given an *l*-mer *M*, the probability that a random string *u* of length *l* has a hamming distance in the range of (*d*_2_,*d*_1_] from *M* is:

9 $$ p_{6} = \frac{{\sum\nolimits}_{i=d_{2}+1}^{d_{1}} {{l}\choose{i}} (|\Sigma|-1)^{i}}{| \Sigma |^{l}}  $$

In one sequence, there are *m*−*l*+1 such *l*-mers. The probability that in such a sequence, there is at least one *l*-mer that is within a hamming distance of *d*_1_ but no *l*-mer that is within a hamming distance of *d*_2_ from *M* is:

10 $$ p_{7} = \sum\limits_{k=1}^{m-l+1} {{m-l+1}\choose{k}} p_{6}^{k} (1-p_{4}-p_{6})^{(m-l+1-k)}  $$

Therefore, the probability that a random *l*-mer out of such dataset meets both (*l,d*_1_) and (*l,d*_2_)-condition is:

11 $$ p_{8} = \sum\limits_{i=1}^{n} p(A_{i} \cap B_{n-i}) = \sum\limits_{i=1}^{n} {{n}\choose{i}} p_{5}^{i} p_{7}^{(n-i)}  $$

In conclusion, the expected number of spurious motifs we can find in the LDDMS model is:

12 $$ |O_{1}| = |O_{2}| = |O_{3}| = | \Sigma |^{l} p_{8}  $$

#### Time complexity of the algorithms

Note that all the three algorithms (LDDMS1, LDDMS2, and LDDMS3) can be nicely parallelized. For LDDMS1, there are parallel versions of LDMS solvers, such as PMS9. For every candidate motif, the checking process is independent and can also be parallelized. For LDDMS2 and LDDMS3, we need to generate the neighnorhood tree for *n*(*m*−*l*+1)*l*-mers out of the input sequences. There are *n*(*m*−*l*+1) independent subproblems and can be assigned to different processors. However, in this paper, we only implement these algorithms sequentially and analyze the time complexity of the sequential versions of these algorithms.

Given a candidate motif of length *l*, checking if it meets (*l,d*_1_) and (*l,d*_2_)-condition in an input of *n* sequences, each of length *m*, will take *O*((*m*−*l*+1)*nl*)=*O*(*mnl*) time. It is easy to see that the brute-force algorithm takes time *O*(|*Σ*|^*l*^*mnl*).

For LDDMS1, qPMS9 can be implemented in *O*(*m*^*k*^*mnN*(*Σ*,*l,d*_1_)) time. *N*(*Σ*,*l,d*_1_) has the same definition as in Eq. 1. *k* is a dynamic variable between 1 and *n*. We get the following:

##### **Theorem 2**

The time complexity of LDDMS1 algorithm is

$$T_{LDDMS1}=O(m^{k}mnN(\Sigma, l, d_{1})+|C_{1}|mnl)$$

where |*C*_1_| is the candidate size of (*l,d*_1_)-motif. An expected number can be obtained from Eq. *5*.

For LDDMS2, in Step 1 and Step 2, generating the neighborhoods from all *l*-mers out of each of the input sequences will take time *O*((*m*−*l*+1)*nN*(*Σ*,*l,d*_2_)). In Step 3, radix sort and removing the duplicates will take time *O*((*m*−*l*+1)*nlN*(*Σ*,*l,d*_2_)). Thus we arrive at:

##### **Theorem 3**

LDDMS2 can be implemented in time

$$ \begin{aligned} T_{LDDMS2}=O \left((m - l + 1)nl N(\Sigma, l, d_{2}) \right) + O(|C_{2}| mnl) \\ = O(mnl N(\Sigma, l, d_{2})+|C_{2}|mnl) \end{aligned} $$

where |*C*_2_| is the candidate size of (*l,d*_2_)-motif. An expected number is given in Eq. *8*.

The following lemma from [16] is useful in computing the time complexity of LDDMS3.

##### **Lemma 1**

For a node *x* in the neighborhood tree, *δ*(*x,i*,*I*) can be updated in *O*(*mn*) time.

##### **Theorem 4**

LDDMS3 can be implemented in time

$$T_{LDDMS3} = O\left(n^{2}m^{2} N\left(\Sigma, l, d_{2}\right)\right) $$

Note this is only the worst-case time complexity and *d*_1_ does not appear in this expression. The actual run time could be much less because a lot of branches can be “pruned”.

## Results and discussion

LDDMS1, LDDMS2 and LDDMS3 are tested on synthetic datasets as well as real datasets. We evaluated our algorithms on a Dell Precisions Workstation T7910 running RHEL 7.0 on two sockets each containing 8 Dual Intel Xeon Processors E5-2667 (8C HT, 20MB Cache, 3.2GHz) and 256GB RAM.

### Synthetic datasets

Following the tradition, we employ combinations of (*l,d*_1_) that are challenging [3]. We vary *d*_2_ from 0 to ⌊*d*_1_/2⌋. The challenging instances of *n*=20,*m*=600 for DNA sequences and the values of *d*_2_ for carrying out the test are listed in Table 1.

**Table 1:** Challenging instances and value of *d*_2_ for test (*n*=20,*m*=600)

*l*	*d*_1_	*d*_2_
7	1	0
8	1	0
9	2	[0,1]
10	2	[0,1]
11	3	[0,1]
12	3	[0,1]
13	4	[0,2]
14	4	[0,2]
15	5	[0,2]
16	5	[0,2]
17	6	[0,3]
18	6	[0,3]
19	7	[0,3]

The challenging instances correspond to a small number of spurious motifs. This will make the candidate size in LDDMS1 very small and hence the time spent in Step 2 in LDDMS1 is trivial. To avoid such problems, we slightly change the way we plant the motifs. We will randomly generate two *l*-mers, *M*_1_ and *M*_2_. The hamming distance of *M*_1_ and *M*_2_ is *q*. Then we insert *M*_1_ into each of the first ⌈*n*/2⌉ input sequences and *M*_2_ into each of the rest ⌊*n*/2⌋ input sequences. A detailed algorithm for generating the test cases is given in Algorithm 4.


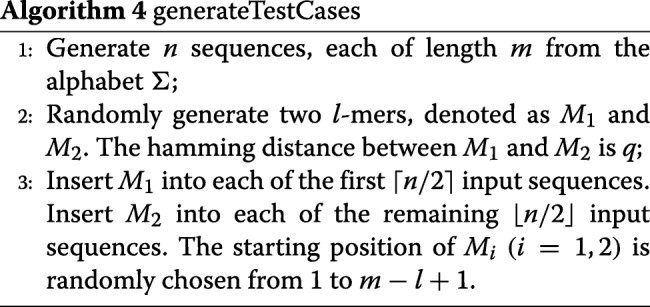


In this way, the common neighbors that are within *d*_2_ hamming distance of *M*_1_ and *M*_2_ are (*l,d*_1_,*d*_2_)-motifs we plant. Generally, when *q* is small, there will be more common neighbors between *M*_1_ and *M*_2_. Conversely, when *q* is large, there are fewer common neighbors between *M*_1_ and *M*_2_. By varying *q*, we can control the output motif size. There is a theory proposed in [8] which proves to be useful here.

#### **Theorem 5**

Two *l*-mers *a* and *b* have a common neighbor *M* such that *Hd*(*a,M*)≤*d*_*a*_ and *Hd*(*b,M*)≤*d*_*b*_ if and only if *Hd*(*a,b*)≤*d*_*a*_+*d*_*b*_.

Applying the above theorem, *q* has to be at a distance of at most 2*d*_2_ for *M*_1_ and *M*_2_ to have common neighbors that are within a *d*_2_ hamming distance. When *d*_2_=0, we set *q*=0, then there will be at least *N*(*Σ*,*l,d*_2_)(*l,d*_1_,*d*_2_)-motifs that can be found. When *d*_2_≠0,*q*=2*d*_2_, there will be at least ${{2d_{2}}\choose {d_{2}}} (l, d_{1}, d_{2})$-motifs that can be found. However, the number of planted (*l,d*_1_)-motifs, i.e., common neighbors that are within a *d*_1_ hamming distance between both *M*_1_ and *M*_2_, is much larger.

We have tested our algorithms on challenging instances of (*l,d*_1_) from (7,1) upto (19,7), where *d*_2_ varies from 0 to ⌊*d*_1_/2⌋. Tables 2, 3 and 4 show the running times of LDDMS1, LDDMS2 and LDDMS3 on different (*l,d*_1_,*d*_2_) values. For small instances such as (*l,d*_1_) = (7,1), (8,1), (9,2), (10,2), LDDMS1 runs faster than LDDMS2 and LDDMS3. This is because qPMS9 is fast and there are only a few (*l,d*_1_)-motifs to check. However, for moderate and relatively large instances, a small value of *d*_2_ will make LDDMS2 run much faster than LDDMS1. For example, for (*l,d*_1_,*d*_2_)=(17,6,1), LDDMS1 takes 29.36 minutes while LDDMS2 only takes 9.19 minutes to solve. However, for large values of *d*_2_, LDDMS2 is slow. Compared to LDDMS2, LDDMS3 performs much better for large instances although it will take more time when *d*_2_ is small. For example, it can solve instances which LDDMS2 cannot solve, such as (*l,d*_1_,*d*_2_)=(18,6,3),(19,7,3).

**Table 2:** Running time of LDDMS1

*d*_2_	0	1	2	3
(*l,d*_1_)				
(7,1)	0.24 s	NA	NA	NA
(8,1)	0.19 s	NA	NA	NA
(9,2)	0.44 s	0.42 s	NA	NA
(10,2)	0.34 s	0.31 s	NA	NA
(11,3)	1.91 s	1.24 s	NA	NA
(12,3)	1.83 s	0.81 s	NA	NA
(13,4)	20.19 s	9.75 s	7.36 s	NA
(14,4)	23.03 s	8.11 s	5.18 s	NA
(15,5)	4.75 m	2.51 m	1.51 m	NA
(16,5)	6.18 m	2.32 m	1.17 m	NA
(17,6)	1.12 h	29.36 m	20.63 m	12.02 m
(18,6)	1.57 h	36.55 m	24.51 m	13.44 m
(19,7)	10.68 h	7.74 h	6.13 h	4.02 h

**Table 3:** Running time of LDDMS2

*d*_2_	0	1	2	3
(*l,d*_1_)				
(7,1)	2.76 s	NA	NA	NA
(8,1)	5.26 s	NA	NA	NA
(9,2)	5.34 s	1.45 m	NA	NA
(10,2)	7.86 s	3.47 m	NA	NA
(11,3)	7.14 s	3.86 m	NA	NA
(12,3)	9.46 s	5.76 m	NA	NA
(13,4)	8.66 s	5.77 m	1.68 h	NA
(14,4)	10.74 s	7.73 m	2.54 h	NA
(15,5)	10.04 s	7.65 m	2.72 h	NA
(16,5)	11.25 s	9.25 m	3.57 h	NA
(17,6)	10.62 s	9.19 m	3.84 h	47.27 h
(18,6)	12.38 s	11.25 m	4.96 h	-
(19,7)	12.13 s	11.54 m	5.52 h	-

**Table 4:** Running time of LDDMS3

*d*_2_	0	1	2	3
(*l,d*_1_)				
(7,1)	5.59 s	NA	NA	NA
(8,1)	6.66 s	NA	NA	NA
(9,2)	8.78 s	8.79 s	NA	NA
(10,2)	9.32 s	9.17 s	NA	NA
(11,3)	12.09 s	12.22 s	NA	NA
(12,3)	11.49 s	11.97 s	NA	NA
(13,4)	16.09 s	16.44 s	25.55 s	NA
(14,4)	14.41 s	14.71 s	19.68 s	NA
(15,5)	20.71 s	21.25 s	35.73 s	NA
(16,5)	18.35 s	18.55 s	28.36 s	NA
(17,6)	25.86 s	26.05 s	53.12 s	8.30 m
(18,6)	24.27 s	23.23 s	40.34 s	6.44 m
(19,7)	32.01 s	33.66 s	1.08 m	7.55 m

Time is in seconds (s), minutes (m) or hours (h). Cells with ‘NA’ indicate instances that are not defined. Cells with ‘-’ imply the algorithm did not complete in the stipulated 48 h

It is obvious that as (*l,d*_1_) instances become larger, all the LDDMS algorithms will take more time. However, an interesting observation is that for a fixed (*l,d*_1_) instance, increasing the value of *d*_2_ will make LDDMS1 run faster but LDDMS2 and LDDMS3 slower. This is because of the way we generate the test cases. If *d*_2_ is very small, then the two *l*-mers we plant will be almost identical. In this case, we will find a lot of (*l,d*_1_)-motifs in the end of Step 2 in LDDMS1. However, small values of *d*_2_ will make the neighborhood tree small, thus LDDMS2 and LDDMS3 will run faster.

### Real datasets

We also used the datesets discussed in [18] to test our algorithms. We chose a group of real datasets. We excluded datasets with only one input sequence because such datasets are not meaningful for our test.

We chose two relatively large number, 18 and 19 for the motif length. Then we re-computed *d*_1_ which will make (*l,d*_1_) challanging instances since each dataset has different number of input sequences and different length for each sequence. However, as we noted before, the challenging instances will make the candidate size in LDDMS1 very small. In this case, we cannot manually plant a motif to avoid such a problem. Therefore, we will increment *d*_1_ by 2. We tested the minimum and maximum number of *d*_2_, i.e., 0 and ⌊*d*_2_/2⌋. Table 5 shows the datasets information and the (*l,d*_1_,*d*_2_) instances we have tested.

**Table 5:** Real datasets from [18]

Datasets	*n*	*m*	*l*	*d*	*d*_1_=*d*+2	*d*_2_
dm01r	4	1500	18	3	5	0, 2
			19	3	5	0, 2
dm03r	3	2000	18	2	4	0, 2
			19	2	4	0, 2
dm04r	4	2000	18	3	5	0, 2
			19	3	5	0, 2
dm05r	3	2500	18	2	4	0, 2
			19	2	4	0, 2

Table 6 shows the running time of LDDMS1, LDDMS2 and LDDMS3 on real datasets. On the real dataset, for fixed (*l,d*_1_), changing *d*_2_ does not affect the running time of LDDMS1 very much. This is because for a real dataset, the candidate size, i.e., the number of (*l,d*_1_) motifs is unchanged. This is also true for the number of (*l,d*_2_) motifs for LDDMS2. Moreover, as one can find, for a fixed *d*_1_, increasing *l* will make LDDMS1 run faster because it will be less challenging. Generally, when *d*_2_ is large, LDDMS2 takes much more time. However, it is hard to say for LDDMS1 and LDDMS3, which one performs better. For example, on real dataset dm05r, when (*l,d*_1_,*d*_2_)=(18,4,2), LDDMS3 (4.07 s) overperforms LDDMS1 (10.79 s). However, on the same dataset, when (*l,d*_1_,*d*_2_)=(19,4,2), LDDMS1 (2.31 s) overperforms LDDMS3 (4.55 s). The actual running time of these algorithms is highly dependent on the dataset and (*l,d*_1_,*d*_2_) values.

**Table 6:** Running time of LDDMS1, LDDMS2, LDDMS3 on real data

Datasets	*l*	*d*_1_	*d*_2_	LDDMS1	LDDMS2	LDDMS3
dm01r	18	5	0	2.04 m	4.15 s	3.42 s
			2	2.03 m	1.61 h	6.67 s
	19	5	0	17.46 s	4.43 s	3.33 s
			2	17.35 s	1.92 h	5.40 s
dm03r	18	4	0	14.80 s	4.64 s	2.73 s
			2	14.73 s	1.78 h	3.49 s
	19	4	0	3.30 s	4.86 s	2.83 s
			2	3.29 s	2.07 h	3.22 s
dm04r	18	5	0	4.37 m	7.16 s	5.89 s
			2	4.37 m	2.83 h	11.17 s
	19	5	0	42.32 s	7.74 s	5.80 s
			2	42.48 s	3.35 h	9.03 s
dm05r	18	4	0	10.70 s	7.03 s	4.07 s
			2	10.79 s	2.68 h	4.77 s
	19	4	0	2.32 s	6.90 s	4.21 s
			2	2.31 s	3.15 h	4.55 s

Time is in seconds (s), minutes (m) or hours (h)

## Conclusions

Efficient motif search algorithms are crucial in solving many bioinformatics problems effectively. In this paper, we have presented the (*l,d*_1_,*d*_2_) motif model, a more general model for the motif search problem. We also have proposed LDDMS1, LDDMS2 and LDDMS3, three exact efficient algorithms to solve the LDDMS problem. Theoretical analysis shows that our algorithms are very competitive. Experimental results also reveal that our algorithms perform well in practice.

In future we will focus on solving harder LDDMS instances, including those involving protein strings. We also plan to implement our algorithms in parallel.
